# The use of Gene-Xpert MTB RIF in the diagnosis of extrapulmonary tuberculosis in childhood and adolescence

**DOI:** 10.1590/0037-8682-0104-2020

**Published:** 2020-10-05

**Authors:** Rafaela Baroni Aurilio, Vivian Vidal Marsili, Thiago da Silva Santos Malaquias, Afrânio Lineu Kritski, Clemax Couto Sant’Anna

**Affiliations:** 1 Instituto de Pediatria e Puericultura Martagão Gesteira, Universidade Federal do Rio de Janeiro, Rio de Janeiro, Brasil.; 2 Instituto de Doenças do Tórax, Universidade Federal do Rio de Janeiro, Rio de Janeiro, Brasil.; 3 Programa Acadêmico de Tuberculose, Instituto de Doenças do Tórax, Faculdade de Medicina, Hospital Universitário Clementino Fraga Filho, Universidade Federal do Rio de Janeiro, Rio de Janeiro, Brasil.; 4 Departamento de Pediatria, Faculdade de Medicina, Universidade Federal do Rio de Janeiro, Rio de Janeiro, Brasil.

**Keywords:** Extrapulmonary, Tuberculosis, Child, Adolescent

## Abstract

**INTRODUCTION:**

Gene-Xpert MTB RIF (Xpert) is based on nucleic acid amplification by real-time polymerase chain reaction, which allows for the identification of *Mycobacterium tuberculosis* and rifampin resistance. We describe the use of Xpert for extrapulmonary tuberculosis (EPTB) in children and adolescents**.**

**METHODS:**

A case series of two reference centers in Rio de Janeiro from 2014-2019.

**RESULTS:**

The final diagnosis of EPTB was established in 11/36 (31%) patients, with five cases detectable by Xpert. For lymph node evaluation (9/11), diagnosis by Xpert occurred in 5/9 patients, all with caseous aspects.

**CONCLUSIONS:**

Xpert can facilitate the rapid diagnosis of lymph node tuberculosis.

Extrapulmonary tuberculosis (EPTB) is caused by secondary hematogenous spread of the primary infection with *Mycobacterium tuberculosis* (*M. tb*.). Bacteriological evidence using traditional methods is infrequent, such as direct smear microscopy and culture for *M. tb*., despite this being the gold standard of TB^1^.

The Gene-Xpert MTB RIF (Xpert) is a test based on the amplification of nucleic acids using real-time polymerase chain reaction (PCR), which allows for identification of *M. tb*. in 2 hours and detection of rifampin resistance (RMP). It can process different materials, but its most excellent applicability is in the sputum of adults with suspected pulmonary TB (PTB)^2^. The positivity in adolescents (16%) was similar to adults in Rio de Janeiro, Brazil^3^. The Ministry of Health of Brazil recommends Xpert in the following specimens: sputum, induced sputum, bronchoalveolar lavage, gastric lavage, cerebrospinal fluid, lymph nodes, and tissue macerates^2^.

Globally, a few studies involving Xpert exclusively in childhood EPTB have been conducted^4^ ^,^ ^5^ ^,^ ^6^. Analysis of Xpert in lymph nodes, including in children and adults in Tunisia, showed detection rates of 77% of cases, demonstrating a rapid diagnosis of lymph node TB (LNTB)^7^. In Tanzania, Xpert analysis of fine-needle aspiration samples in pediatric patients with symptoms suggestive of LNTB showed a 19% positivity rate^4^. The meningeal form, due to its severity and lethality, requires early diagnosis, for adequate therapy. Xpert may be a superior tool to acid-fast bacilli (AFB) smears in these cases^6^.

A systematic review to access the accuracy of Xpert in non-respiratory samples for pulmonary TB and EPTB in children and adults identified a sensitivity of 98% (95% confidence interval [CI], 87-99%), compared with concurrent culture, in several tissues^8^. 

This article aims to describe the use of Xpert in EPTB in children and adolescents in two reference centers for pediatric TB.

This is a case series of the Municipal Hospital Raphael de Paula e Souza (HMRPS) and Instituto de Puericultura e Pediatria Martagão Gesteira (IPPMG) situated in the city of Rio de Janeiro, from 2014-2019. The IPPMG Ethics Committee approved the work. Patients aged <19 years with suspected EPTB, who attended the outpatient or were hospitalized in the respective hospitals, and whose collected specimens were submitted to Xpert and other diagnostic methods were included. The variables studied were age, sex, history of contact with a patient with pulmonary TB in the last 2 years, tuberculin skin test (TST) (positive ≥5 mm and negative <5 mm), Xpert (detectable and undetectable), AFB (positive and negative), culture for *M. tb.* (positive and negative), and histopathological (findings of chronic granulomatous disease and necrosis, with/without caseous material, in lymph node biopsy). The highest probability of EPTB from different locations was made by the doctors who treated the patients in both institutions. The final diagnosis of EPTB was established, after a favorable clinical response within 30 days from the beginning of anti-TB therapy, without the use of other antimicrobials.

Thirty-six patients with symptoms suggestive of EPTB were studied. The final diagnosis of EPTB was established in 11/36 (31%) patients, and the other 25 (69%) received other diagnoses. No patient had a history of contact with tuberculosis.

The Xpert result and final diagnosis of EPTB are described in Figure 1, and the description of the cases with a final diagnosis of TB are listed in Table 1.

**FIGURE 1:**
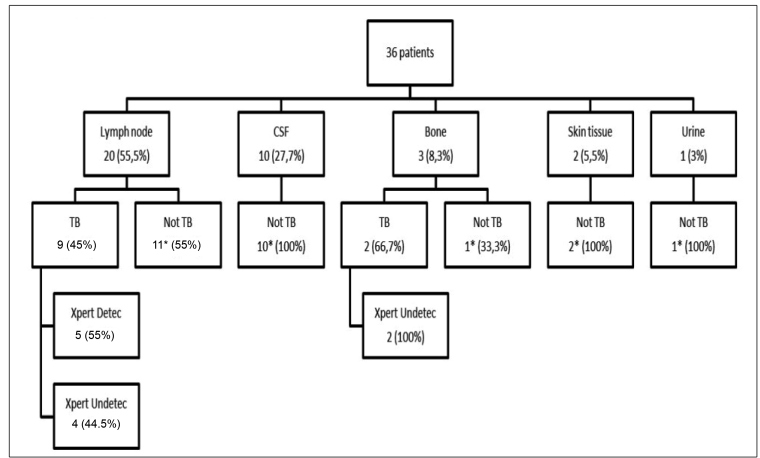
Final diagnosis in 36 patients with EPTB signs and symptoms submitted to Xpert. **EPTB:** extrapulmonary tuberculosis; **TB:** tuberculosis; **Detec**: detectable; **Undetec**: undetectable; **CSF**: cerebral spinal fluid. *Xpert undetectable.

**Table:** 

Case number	Age (years)	TST	Specimen	Xpert	AFB	Culture	Histopathological
**1**	0.6	Neg	Cervical LNTB	Detec	Neg	Pos#	TB
**2**	0.8	Neg	Supraclavicular LNTB	Detec	Neg	Pos	ND
**3**	0.8	Neg	Axillar LNTB	Detec	Pos	Pos#	TB
**4**	1	Neg	Cervical LNTB	Detec	Neg	Pos	TB
**5**	3.2	Pos	Inguinal LNTB	Undetec	Neg	Neg	TB
**6**	7	Pos	Inguinal LNTB	Detec	Neg	Neg	TB
**7**	11.1	Pos	Cervical LNTB	Undetec	Neg	Neg	TB
**8**	12.8	Pos	Inguinal LNTB	Undetec	Neg	Neg	TB
**9**	14.9	Pos	Cervical LNTB	Undetec	Neg	Pos*	TB
**10**	1.1	Pos	Bone	Undetec	Neg	Neg	TB
**11**	11	Neg	Bone	Undetec	ND	Pos*	TB

TST: Tuberculin Skin Test; Pos: positive; Neg: negative; Undetec: Undetectable; Detec: detectable; ND: not done; TB: tuberculosis; LNTB: Lymph node tuberculosis.*Antimicrobial drug sensitivity test = multi-resistence (MR); # Antimicrobial drug sensitivity test = sensitivity to first-line drugs.

In the present study, of the 11 patients with a final diagnosis of EPTB, 5 had detectable Xpert, which resulted in a faster diagnosis than culture and histopathological examination. If we only evaluated lymph node samples, the diagnosis by Xpert occurred in more than half of the patients, and all had a caseous aspect detected during biopsy. A similar result was observed in Ethiopia^9^ in 152 adults and children with symptoms suggestive of LNTB, with an Xpert sensitivity of 78% (95% CI, 73.7-83.3%) and specificity of 64% (95% CI, 69.4-78.6%) using culture as the gold standard. In addition, Xpert detected more than half of the samples with a caseous aspect, which indicates a chronic lesion with a sizeable bacillary population. However, there was no contribution to the detection of RMP resistance by Xpert in our study. In patients with a final diagnosis of LNTB multidrug resistance, the final diagnosis was detected using culture, a method that can take up to 60 days, with a false-negative by Xpert. In the study by Bholla et al^4^, when analyzing children with probable TB lymphadenitis, they also observed false-negative results from Xpert. Of 9 patients with a positive culture for TB, 5 were positive by Xpert, but resistance to RMP using this method was not the objective of this study.

In our study, there was no positivity for Xpert in the skin, bone, and urine. 

Likewise, our data did not show positivity for Xpert in cerebrospinal fluid. On the other hand, this diagnosis was not established in 10 patients with suspected tubercular meningitis. In a study carried out in India^6^with 28 children aged 2 months to 12 years with signs suggestive of TB, the positivity of Xpert in the pediatric age group was 21.4%.

In our study, only one patient was suspected of urinary tract TB, the Xpert result was negative, and the final TB diagnosis was not established. The use of Xpert for the diagnosis of urinary tract TB in adults has shown good sensitivity (82.7%; 95% CI, 69.6-91.1%) and specificity (98.7%; 95% CI, 94.8-99.7%)^10^. Lopez^5^ et al., who simultaneously analyzed Xpert in urine and respiratory samples from children with symptoms suggestive of PTB, sought the detection of *M. tb.* in urine as a reflex of PTB. The authors have shown that the use of Xpert in urine did not contribute to the diagnosis of PTB in this age group. 

EPTB, generally, shows no reactivity to TST; however, in the present study, most patients had a positive result. TST data is relevant for the diagnosis of these TB forms^1^.

The present study had some limitations, such as a small sample of different extrapulmonary specimens, making it challenging to compare Xpert performance between them and preventing the calculation of the method accuracy. Additionally, this study involved only pediatric patients (with supposed low bacillary charge of *M. tb.*), which may explain the low positivity of Xpert. However, the correlation of Xpert with the caseous appearance in patients with LNTB occurred in all cases.

We demonstrate that Xpert can contribute to the investigation of childhood EPTB, more specifically in the lymph nodes, providing rapid diagnosis compared with culture and histopathology.
